# Species-Specific Marker Discovery in Tilapia

**DOI:** 10.1038/s41598-019-48339-2

**Published:** 2019-09-10

**Authors:** Mochamad Syaifudin, Michaël Bekaert, John B. Taggart, Kerry L. Bartie, Stefanie Wehner, Christos Palaiokostas, M. G. Q. Khan, Sarah-Louise C. Selly, Gideon Hulata, Helena D’Cotta, Jean-Francois Baroiller, Brendan J. McAndrew, David J. Penman

**Affiliations:** 1 Institute of Aquaculture, University of Stirling, Stirling, Scotland United Kingdom; 2 Program Study of Aquaculture, Agriculture Faculty, Universitas Sriwijaya, South Sumatra, Indonesia; 3 Max Planck Institute of Psychiatry, 80804 Munich, Germany; 4 Department of Fisheries Biology and Genetics, Bangladesh Agricultural University, Mymensingh, Bangladesh; 5 Institute of Animal Science, Agricultural Research Organization, The Volcani Center, Rishon LeZion 7505101, Israel; 6 ISEM, Univ. Montpellier, CNRS, IRD, EPHE, Montpellier, France; 7 CIRAD, Campus Int. Baillarguet, 34398 Montpellier, France

**Keywords:** Genetic markers, Genetic variation

## Abstract

Tilapias (family Cichlidae) are of importance in aquaculture and fisheries. Hybridisation and introgression are common within tilapia genera but are difficult to analyse due to limited numbers of species-specific genetic markers. We tested the potential of double digested restriction-site associated DNA (ddRAD) sequencing for discovering single nucleotide polymorphism (SNP) markers to distinguish between 10 tilapia species. Analysis of ddRAD data revealed 1,371 shared SNPs in the *de novo*-based analysis and 1,204 SNPs in the reference-based analysis. Phylogenetic trees based on these two analyses were very similar. A total of 57 species-specific SNP markers were found among the samples analysed of the 10 tilapia species. Another set of 62 species-specific SNP markers was identified from a subset of four species which have often been involved in hybridisation in aquaculture: 13 for *Oreochromis niloticus*, 23 for *O*. *aureus*, 12 for *O*. *mossambicus* and 14 for *O*. *u*. *hornorum*. A panel of 24 SNPs was selected to distinguish among these four species and validated using 91 individuals. Larger numbers of SNP markers were found that could distinguish between the pairs of species within this subset. This technique offers potential for the investigation of hybridisation and introgression among tilapia species in aquaculture and in wild populations.

## Introduction

The tilapias are a group of African and Middle Eastern cichlid fish, with more than 70 species^1,2^. Tilapias, mostly from the *Oreochromis* genus, are cultured in over 120 countries^3^, and global production reached 5.6 million tonnes in 2015^4^. Hybridisation and introgression among tilapia species (mostly within genera) has occurred widely due to anthropogenic impacts. Hybridisation and introgression in aquaculture has been intentional in some cases (*e*.*g*. the widely used *O*. *niloticus* × *O*. *aureus* F_1_ hybrid^5^) and accidental^6^ in others (*e*.*g*. introgression of *O*. *mossambicus* into farmed *O*. *niloticus* stocks in Asia^6^). Introgression has occurred in a range of natural or semi-natural habitats: *e*.*g*. between *T*. *zillii* and *T*. *guineensis* following damming of a river in the Ivory Coast to form the man-made Lake Ayame^7^; between introduced *O*. *niloticus* and native *O*. *esculentus* in Lake Victoria, eventually leading to loss of native species^8^; and between *O*. *mossambicus* and *O*. *niloticus* in Southern Sri Lanka – both introduced, outside of their native ranges^9^. Distinguishing tilapia species, hybrids and introgressed populations is of importance for both farmed and wild populations.

Since reproductively viable hybrids (and further generations) have often resulted from interspecies crosses in tilapias, external morphometrics is of limited use for distinguishing tilapia species and hybrids^10^. The identification of many tilapia species, both wild and farmed, has also become more difficult with the extensive introduction of tilapia species outside of their natural ranges. Therefore, genetic markers offer a more reliable means to resolve the genetic composition of established, feral hybrids in new environments, and the composition of mixed species in culture^11^. Different marker technologies have been applied to study hybridisation and introgression, such as allozymes^12–15^, microsatellite markers^16,17^, Randomly Amplified Polymorphic DNA (RAPD)^18–20^ and restriction fragment length polymorphisms (RFLPs) in ribosomal DNA^21,22^. While mitochondrial DNA (mtDNA) analysis has been used to distinguish tilapia species^23,24^, it is of limited use in analysing hybridisation and introgression. The nuclear DNA markers used to date have limitations, the most important being the small number of markers available that distinguish between tilapia species (*e*.*g*. species-specific alleles have been found using allozymes, but only for a few loci^12^). Multiple diagnostic markers are required to analyse species composition in introgressed populations. To date no study has used single-nucleotide polymorphism (SNP) to identify tilapia species.

High-throughput sequencing technologies make it possible to carry out genotyping-by-sequencing in many species using many individuals, whether reference genomes are available or not. The objective of the research described here was to test the potential of double digest restriction-site associated DNA^25^ (ddRADseq) for discovering SNP markers to distinguish between 10 tilapia species (including two sub-species of *O*. *niloticus*) and analyse the distribution of such markers in the genome. Based on the ddRADseq results, a panel of 24 candidate species-specific SNP markers for four tilapia species was selected and validated against a wider selection of fish using Kompetitive Allele Specific PCR (KASP) assays.

## Results

### COI sequencing

**Figure 1:**
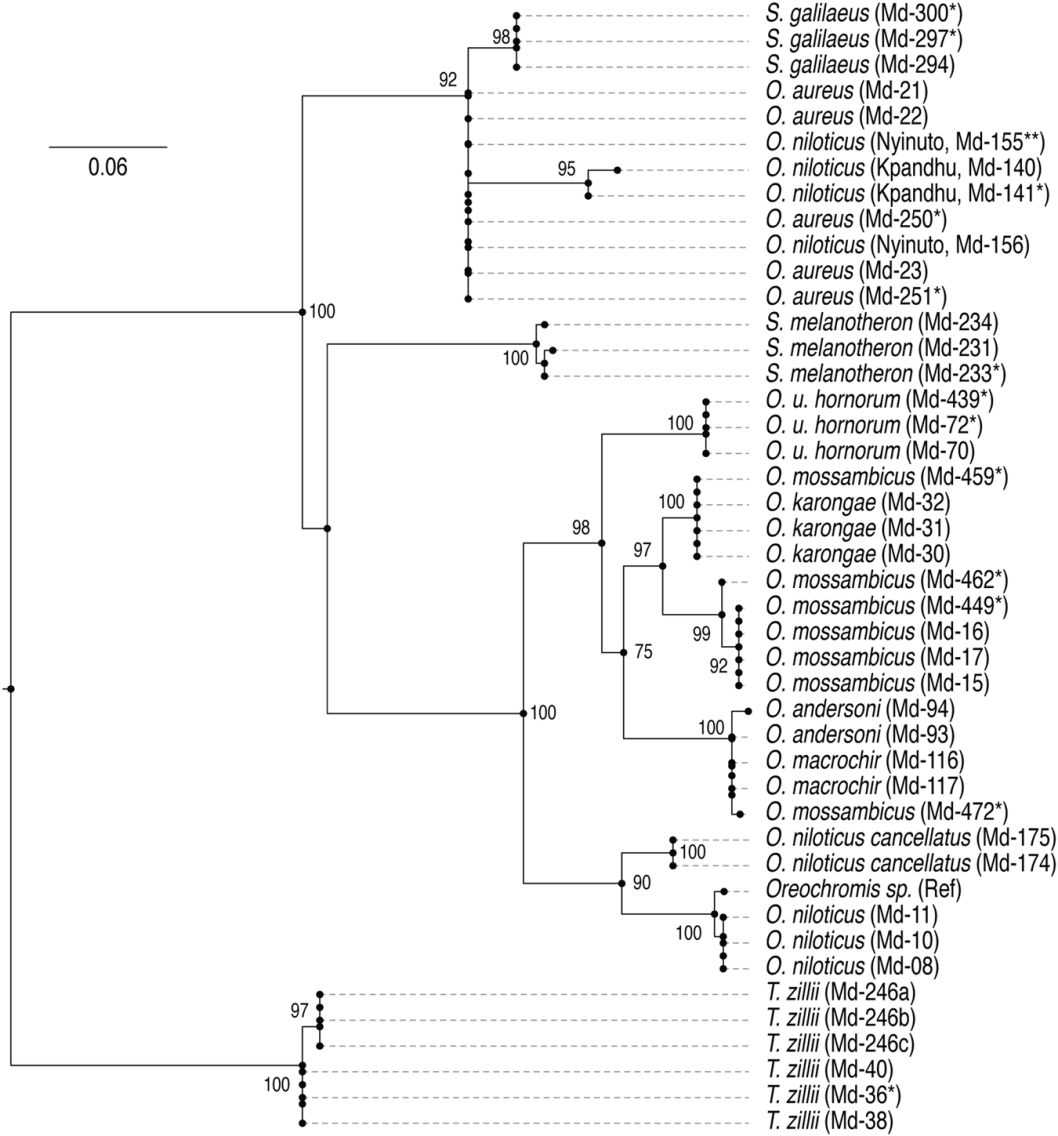
A gene tree of tilapia species inferred from COI sequences, and rooted to *T*. *zillii*. All sequences were aligned, and the tree was constructed using RAxML. *Samples not used for ddRAD seq. **Sample also used in KASP validation.

The retrieved cytochrome oxidase subunit I (COI) partial sequences of the tilapiine species (Table 1) varied between 395–631 bp, and matched those in the Barcode of Life Data System (BOLD) and the NCBI GenBank Database. The COI gene tree separated the *Tilapia* genus from the other two genera, however *Sarotherodon* and *Oreochromis* were not clearly separated (Fig. 1). The largest group consisted of most of the *Oreochromis* species *i*.*e*. *O*. *niloticus*, *O*. *mossambicus*, *O*. *karongae*, *O*. *u*. *hornorum*, *O*. *andersonii* and *O*. *macrochir* (the last two were not separated from each other within this group). However, *O*. *aureus* and some *O*. *niloticus* were in a group with *S*. *galilaeus*, while *S*. *melanotheron* was in a separate group from *S*. *galilaeus*. West African *O*. *niloticus* (Onn_Kp and Onn_Ny) exhibited COI haplotypes typical of *O*. *aureus*, as previously reported^26^, although nuclear markers clearly indicated the differences between these two species.

**Table 1:** Origin of tilapia samples used in ddRAD analysis, SNP validation by KASP and COI sequencing.

Species	Acronym	Strain/Population	ddRAD	KASP	COI	Sampling origin
*O*. *niloticus niloticus*	Oni	Stirling	6	14	5	Lake Manzala, Egypt
		Kpandu	12	8	2	Ghana
		Nyinuto	12	8	2	Ghana
*O*. *niloticus cancellatus*		Hora	13	0	2	Ethiopia
		Koka	12	0	0	Ethiopia
		Metahara	8	0	0	Ethiopia
*O*. *mossambicus*	Omo	Stirling	5	7	3	Zimbabwe
		Natal	10	8	1	South Africa
		Singapore	0	7	0	Unknown
		Eastern Cape	0	0	2	South Africa
		Western Cape	0	0	1	South Africa
*O*. *aureus*	Oau	Stirling	5	8	1	Lake Manzala, Egypt
		Ein Feskha	10	15	2	Israel
*O*. *karongae*	Oka	Stirling	5	0	3	Lake Malawi
*O*. *u*. *hornorum*	Oho	Israel	5	16	2	Tanzania
		Stirling	0	0	1	Unknown
*T*. *zillii*	Tzi	Stirling	5	0	3	Lake Manzala, Egypt
		Ghana	5	0	3	Ghana
*S*. *galilaeus*	Sga	Israel	5	0	3	Israel
*O*. *andersonii*	Oan	Itezhi-tezhi	6	0	2	Zambia
*O*. *macrochir*	Oma	Itezhi-tezhi	4	0	2	Zambia
*S*. *melanotheron*	Sme	Ghana	4	0	3	Ghana
**Total**			**132**	**91**	**43**	

A total of 34 samples (10 *O*. *niloticus*, 6 *O*. *mossambicus*, 13 *O*. *aureus* and 5 *O*. *u*. *hornorum*) were analysed using both ddRADseq and KASP. The 43 samples analysed by COI sequencing comprised representatives from each of the ten-fish species (of which 29 samples were also characterised by ddRAD).

### Double digest RAD library sequencing

**Figure 2:**
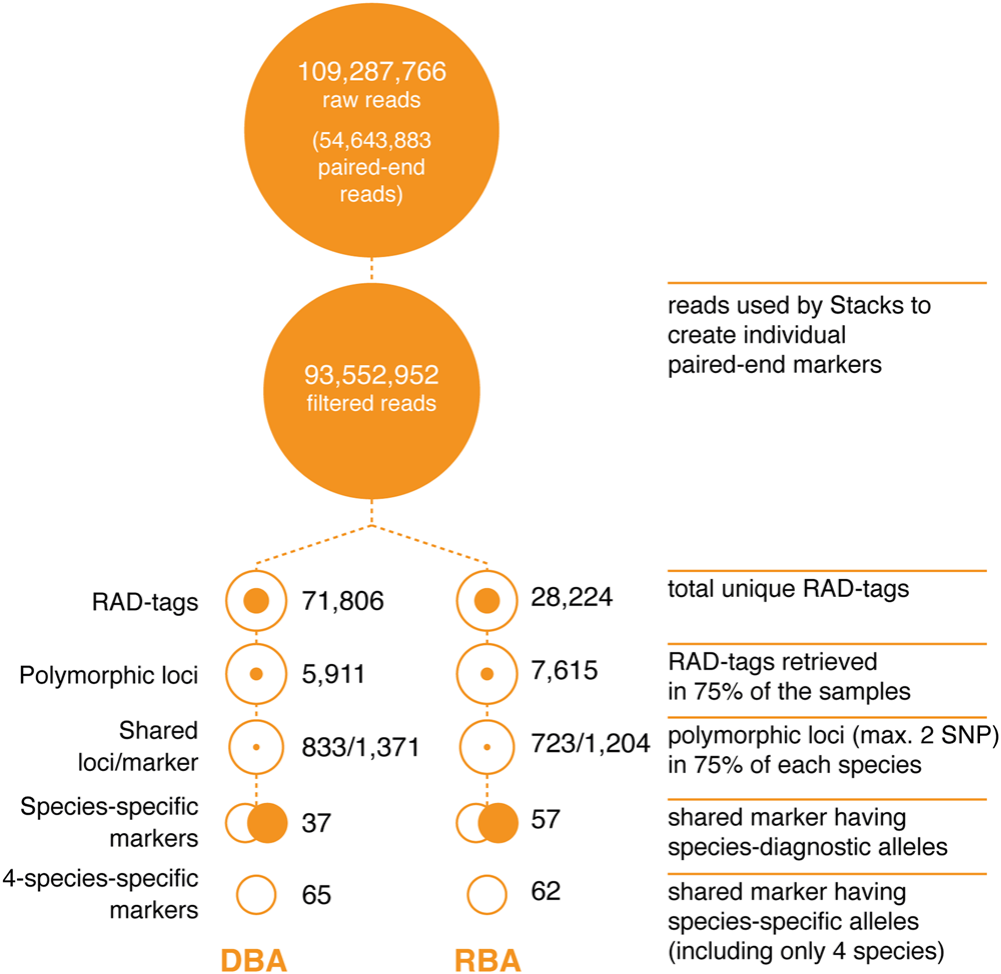
The number of reads, loci and markers for the *de novo*-based analysis (DBA) and reference genome-based analysis (RBA).

In total, 54,643,883 paired-end raw reads were produced from the three ddRAD libraries. After removing low quality sequences, ambiguous barcodes and orphaned paired-end reads, 85.6% of the raw reads were retained. In total, the Stacks analysis identified 71,806 unique RAD-tags (*i*.*e*., the total number of loci across all species, with overlapping subsets of loci among species) in the *de novo*-based analysis (DBA) and 28,224 unique RAD-tags in the reference genome-based analysis (RBA) (Fig. 2 and Supplementary Table S1).

### Sequence analysis

**Figure 3:**
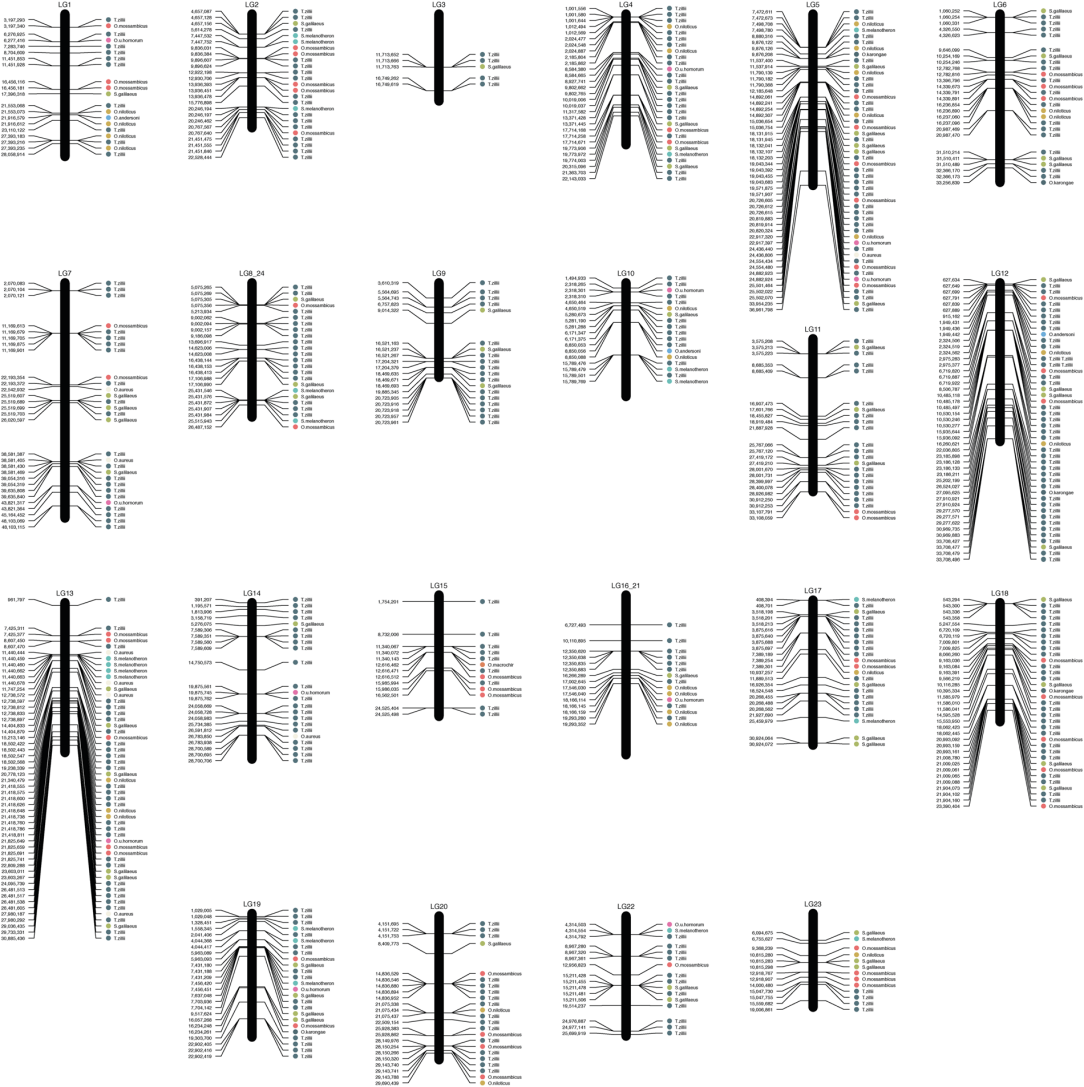
Physical mapping of 644 species-specific SNP markers (RBA, with up to five SNPs per ddRAD locus – see text for further details) in the reference genome.

A total of 5,911 and 7,615 shared loci (*i*.*e*., biallelic RAD-tags with one or more SNPs and present in at least 75% of the samples) were found in the DBA and RBA respectively, and were used in subsequent analyses and for the phylogenetic reconstruction. Species-specific loci were identified as the RAD-tags exhibiting no intraspecific polymorphism but showing interspecific polymorphism (*i*.*e*., fixed differences between species so that one species had one allele that differed from all other species) and present in at least 75% of each taxon: 235 loci (427 markers) and 363 loci (644 markers) in DBA and RBA, respectively (Table 2). Species-specific loci with only one or two SNPs were selected (to facilitate the development of polymerase chain reaction [PCR]-based SNP assays), resulting in a subset of 37 (from 30 loci) and 57 (from 47 loci) markers from DBA and RBA, respectively. Physical mapping of the 644 species-specific markers (RBA) in the reference *O*. *niloticus* genome (Fig. 3) suggested that the species-specific markers were distributed randomly across the genome.

**Table 2:** Number of species-specific SNPs identified in loci with up to five species-specific SNPs per locus.

SNPs/locus	Species									
	Oni	Omo	Oau	Oho	Oan	Oma	Oka	Sme	Sga	Tzi
1	1/1	0/0	0/0	0/0	0/0	0/0	1/1	0/0	3/3	0/3
1–2	1/1	2/4	0/0	1/1	0/0	0/0	1/1	1/3	6/8	25/39
1–3	4/6	11/16	0/1	3/6	1/1	0/0	2/2	1/7	11/19	67/101
1–4	10/15	19/29	1/3	6/8	1/3	0/0	4/5	4/12	21/33	142/214
1–5	19/32	47/63	7/11	8/11	2/4	0/1	5/5	14/26	45/67	280/424

Numbers shown are for DBA/RBA respectively (see text for further details).

### SNP-based phylogenetic tree reconstruction

**Figure 4:**
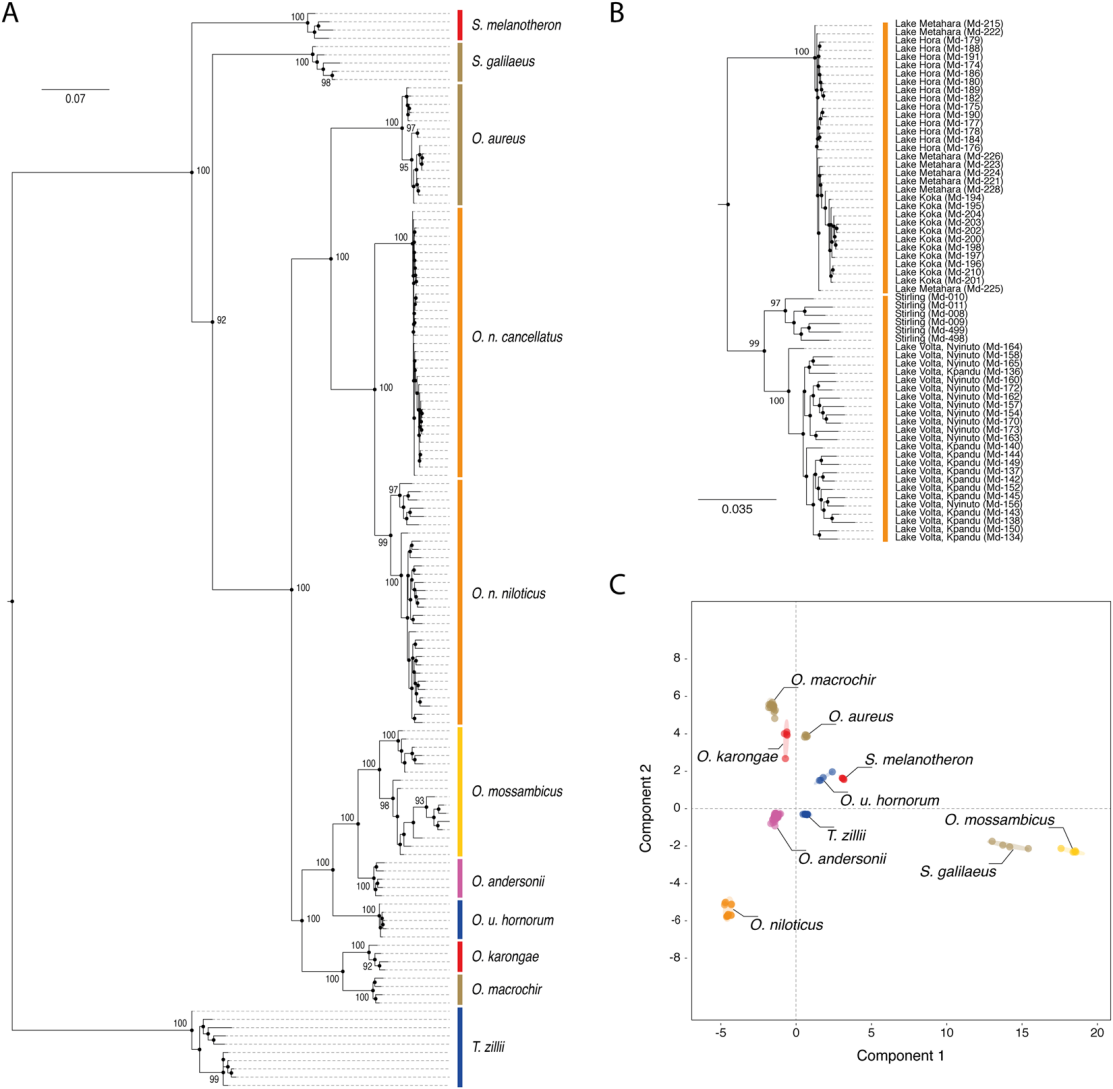
The discriminant ability of RAD markers. (**A**) Phylogenetic tree of tilapia species inferred from 1,204 shared markers from reference-based analysis (RBA) and rooted to *T*. *zillii*. All sequences were aligned, and the tree was constructed using RAxML. The best ML bipartition tree is provided with bootstrap support when higher than 75%. (**B**) An enlarged version of the phylogenetic tree shown, encompassing the two subspecies of *O*. *niloticus*. (**C**) Principal Component Analysis of the 1,204 shared markers.

The phylogeny of the tilapiine species was inferred from all shared loci in the DBA and RBA which contained a maximum of two SNPs. A total of 1,371 SNP markers in 833 loci were identified across all tilapia species based on DBA, while 1,204 shared markers in 723 loci were obtained based on RBA. Of these, 721 loci were common to the DBA and RBA, while 112 loci were DBA-specific and two were RBA-specific. The phylogenetic trees derived from DBA (Supplementary Fig. S1) and RBA (Fig. 4A) were very similar. These phylogenetic analyses showed *T*. *zillii* furthest from all other tilapia species, with *Sarotherodon* spp. (*S*. *melanotheron* and *S*. *galilaeus*) closer to *Oreochromis* spp. (*O*. *niloticus*, *O*. *aureus*, O. *mossambicus*, *O*. *karongae*, *O*. *u*. *hornorum*, *O*. *macrochir* and *O*. *andersonii*). The support values across the branches gave a high level of confidence for species discrimination. An enlargement of the *O*. *niloticus* clade (Fig. 4B) showed that there was no clear difference among fish from Lakes Hora, Koka and Metahara within the subspecies *O*. *niloticus cancellatus*. In the subspecies *O*. *n*. *niloticus*, the Stirling stock (Egyptian origin) can be distinguished from the Volta drainage samples, but there was no discrimination between the two samples (Nyinuto and Kpandu) from the latter.

To better capture the discriminant ability of these markers a principal component analysis (PCA) was conducted from the 1,204 shared SNP markers from the RBA, using *R/adegenet* (Fig. 4C). Ten distinct species-specific clusters were separated using the first two components (78.2% of cumulative variance).

### Species-specific markers for aquaculture species

When the set of species being compared was reduced to the four *Oreochromis* species which have commonly been hybridised in aquaculture, more (65/62 DBA/RBA) species-specific markers were identified (with a maximum of two SNPs per locus allowed): *O*. *niloticus* (14/13 markers), *O*. *aureus* (26/23), *O*. *mossambicus* (14/12) and *O*. *u*. *hornorum* (11/14). These markers enabled each species to be distinguished from the other three. Subspecies-specific markers were also found in *O*. *niloticus*. Three markers distinguished between sub-species *O*. *n*. *niloticus* and *O*. *n*. *cancellatus*, representing three natural geographical regionss *i*.*e*. Nilotic, Awash system and Sudano-Sahelian region.

### SNP assay validation

Twenty-four of these putative species-specific KASP markers were designed and tested. These markers were distributed across 11 of the Nile tilapia linkage groups (Supplementary Fig. S2: n = 1–5 markers per LG). Fifteen were selected to be species-specific in the set of four species based on the ddRADseq data, while eight SNPs (*Oni*9497, *Omo*10818, *Omo*3481, *Omo*3582, *Omo*4092, *Omo*7956, *Omo*2007 and *Omo*8084) and the single *Oau*4411 SNP were predicted only to clearly distinguish between three species with the exception of *O*. *u*. *hornorum* and *O*. *niloticus* respectively. Individual genotypes for each of the 24 markers tested by KASP are listed in Supplementary Table S2 and the allele frequencies are summarised in Table 3. Comparison of genotypes generated by KASP assays with ddRADseq data, based on 34 individuals and 24 SNPs, with 22 genotypes missing from the ddRADseq data, showed 99.4% match (789/794). In all five mismatches, the KASP assays indicated heterozygotes whereas the ddRADseq data indicated homozygotes.

**Table 3:** Summary of diagnostic allele frequency for 24 putative diagnostic markers identified by ddRAD and tested by KASP genotyping assays in 91 individuals to distinguish among *O*. *niloticus*, *O*. *mossambicus*, *O*. *aureus* and *O*. *u*. *hornorum*.

Diagnostic for	Frequency of putative diagnostic allele (%)					
	Marker name	Diagnostic SNP	*O*. *niloticus* (n = 30)	*O*. *mossambicus* (n = 22)	*O*. *aureus* (n = 23)	*O*. *u*. *hornorum* (n = 16)
*O*. *niloticus*	*Oni*3057	A	100*	0	0	0
	*Oni*9497	T	100*	0	0	31
	*Oni*5782	A	100*	0	0	0
	*Oni*2675	G	97*	0	0	0
	*Oni*1276	A	97*	0	0	0
*O*. *mossambicus*	*Omo*2657	A	0	100*	0	0
	*Omo*10818	C	0	100*	0	100
	*Omo*3481	C	0	100*	0	100
	*Omo*3582	A	0	100*	0	100
	*Omo*4092	C	0	100*	0	100
	*Omo*7956	T	0	100*	0	100
	*Omo*2007	A	0	100*	0	100
	*Omo*10120	A	0	95*	0	0
	*Omo*8084	G	0	91*	0	56
*O*. *aureus*	*Oau*9418	C	0	0	100*	0
	*Oau*8029	G	0	0	100*	0
	*Oau*966	T	0	0	100*	0
	*Oau*3873	C	0	0	100*	0
	*Oau*3001	A	3	0	100*	0
	*Oau*2890	T	12	0	100*	0
	*Oau*4411	T	5	0	100*	0
*O*. *u*. *hornorum*	*Oho*4270	A	0	0	0	100*
	*Oho*10793	G	0	0	0	100*
	*Oho*10199	A	0	0	0	100*

Refer to Supplementary Table S3 for full genotype data. *Frequencies of the putative diagnostic alleles in the target species.

Of the subset of 15 markers designed to discriminate all four species, 10 were still found to be 100% specific for the selected test panel of 91 fish representing the four species. This included two *O*. *niloticus* markers (*Oni*3057, *Oni*5782), one *O*. *mossambicus* marker (*Omo*2657), four *O*. *aureus* markers (*Oau*9418, *Oau*8029, *Oau*966, *Oau*3873) and all three *O*. *u*. *hornorum* markers tested (*Oho*4270, *Oho*10793, *Oho*10199). Another three (*Oni*2675, *Oni*1276 and *Omo*10120) were species-specific apart from the target species showing a low frequency (<5%) of the alternate allele in the test panel. The remaining two *O*. *aureus* markers (*Oau*3001, *Oau*2890) showed a low frequency (1–5%) of the putative diagnostic allele in the non-target *O*. *niloticus* species. Of the nine markers designed to distinguish between three species, eight were found to be 100% specific in the test panel, with as predicted *O*. *u*. *hornorum* sharing a common allele with the target species of 2–23% frequency. The final marker *Omo*8084 designed to separate the three species *O*. *mossambicus* from *O*. *niloticus* and *O*. *aureus* showed polymorphism both by the low frequency of the alternate allele in the target species (9%) and the diagnostic allele in *O*. *u*. *hornorum (*13%).

## Discussion

Inferring a comprehensive genetic structure of SNP variation in wild and farmed populations of tilapia species would require the sampling of tens of species with hundreds of individuals each. There is a trade-off between sample size and number of markers (SNPs in this case), which allows to reduce the sample size per species, sub-species or population to some extent. In addition, adding more than one geographic sample per species, where possible, strengthened the analysis. In the present study, we analysed fish from multiple populations of the main aquaculture species, plus representative species to cover the three main genera. This gave a sufficiently wide sample set to explore the use of ddRADseq for species discrimination and phylogenetic analysis. It was not possible to obtain samples from more than one population for several of the species concerned.

The phylogenetic trees developed from shared SNP markers showed significant separation between the three genera of tilapia: *Tilapia*, *Sarotherodon* and *Oreochromis*. These trees showed *T*. *zillii* as the most distant, followed by *S*. *galilaeus* and *S*. *melanotheron*, while the *Oreochromis* species were in a third distinct clade. There were two different clusters within *Oreochromis*, *i*.*e*., *O*. *niloticus* and *O*. *aureus* were separated from *O*. *macrochir*, *O*. *karongae*, *O*. *andersonii*, *O*. *mossambicus* and *O*. *u*. *hornorum*.

Unlike the ddRADseq-based analyses, the COI gene tree did not clearly separate the *Sarotherodon* and *Oreochromis* genera (in contrast to the ddRADseq-based analyses, in which all three genera were separated). In addition, the COI sequence data did not separate *O*. *andersonii* and *O*. *macrochir* or West African *O*. *niloticus* from *O*. *aureus*. Although nuclear markers (allozymes) showed distinct separation between *O*. *aureus* and *O*. *niloticus* in West African populations, the same mtDNA sequences were detected in both species^26^. The current study indicates a very clear differentiation between these two species at the nuclear DNA level. Furthermore, despite overlapping natural distributions of *O*. *aureus* and *O*. *niloticus*^2^, they do not interbreed in nature^27^. Considering that the COI gene tree is based on a single maternally inherited locus, it is not surprising that it did not have the depth of the trees constructed from multiple nuclear DNA markers.

Earlier studies emphasise the contrast between tilapia phylogenies developed from nuclear markers (allozymes^15,28^; five nuclear DNA markers^1^) and mtDNA sequence^1,23,29^. One study did integrate data from five nuclear DNA markers and mtDNA in a revised taxonomy of tilapias^1^, based on a broader range of species than the present study. A major drawback of earlier studies was the limited number of markers on which they were based. It seems likely that phylogenies based on hundreds of nuclear markers derived from ddRADseq (or other types of genotyping by sequencing), as in the present study, should produce robust results to resolve such relationships.

Phylogenetically, *O*. *andersonii* could be distinguished from *O*. *macrochir* using nuclear SNP data but not using the COI sequence in our study. A report showed evidence of hybridisation and introgression of these two (native) species with introduced *O*. *niloticus* in the Kafue River fishery (based on eight microsatellite loci), resulting in a complex mixed population consisting of genetic material from all three species^30^. The introduced *O*. *niloticus* presumably eliminated the behavioural isolation mechanism^31^ between these two native species^30^. In addition, a low frequency of hybridisation occurred between the introduced *O*. *niloticus* and *O*. *andersonii* with native *O*. *mossambicus* in South Africa^8^. The success of SNP markers to discern four species can be discerned based on the shared SNP markers (Figs 3 and 4), which is promising for the use of such SNP markers in future studies involving potential hybridisation and introgression among these and other groups of tilapia.

The shared SNP markers also showed a clear distinction between the two sub-species of *O*. *niloticus*, *O*. *n*. *cancellatus* and *O*. *n*. *niloticus*, which formed two separate clades in the phylogenetic analysis. The *O*. *n*. *cancellatus*, found in Lakes Hora, Koka and Metahara, all in the Awash System in the Ethiopian Rift Valley, formed a single branch with little discrimination among the three lake populations studied.

Reference genome-based analysis of the ddRADseq data identified 57 species-specific SNP markers among ten tilapia species when a maximum of two SNPs per locus was allowed (to facilitate KASP assay design). The analysis found species-specific markers from seven species, but none were found for *O*. *andersonii*, *O*. *aureus or O*. *macrochir*. However, when the number of SNPs per locus criterion was relaxed to a maximum of five, species-specific markers were identified for all ten species. The species-specific markers from loci having 1–2 SNPs could be genotyped using single SNP assays, while those containing more SNPs would require other methods of analysis, *e*.*g*. PCR and sequencing.

It would be desirable to have a simple technique to distinguish between the species in the *Oreochromis* genus that are commonly used in aquaculture. Analysis of the ddRADseq dataset allowed the development of PCR-based assays for several SNP markers that served this purpose. Only twenty-four were screened in this way; larger numbers could be developed, particularly if the requirement was only to compare pairs of species. For instance, between *O*. *niloticus* and *O*. *mossambicus*, both of which are commonly found in Asian countries in aquaculture and ferally, there were 66 SNP markers at 60 loci that could be used for species discrimination.

Validation of individual species-specific SNPs using PCR-based KASP assays revealed a very high level of agreement with the genotypes from the ddRADseq data, and would allow promising SNPs to be assayed more cheaply and/or in laboratories without access to a sequencer, broadening the potential for application to a wide range of situations where hybridisation/introgression are suspected in aquaculture or wild populations. The availability of pure species control samples is important. In the few cases where there was a mismatch, the KASP assay allowed better discrimination of heterozygotes. Under representation of heterozygotes is a known inherent bias of RAD-seq methodologies^32^.

Analysis of fixed allele patterns in the ddRADseq dataset also identified three potential subspecies-specific SNP markers in *O*. *niloticus*. These markers distinguished the sub-species *O*. *n*. *niloticus* and *O*. *n*. *cancellatus*, based on Egyptian and West African *O*. *n*. *niloticus*, and *O*. *n*. *cancellatus* from three different sources in Ethiopia^33^. One of the SNPs was located in LG1 (marker id_5516A), with the allele A in *O*. *n*. *niloticus* and G in *O*. *n*. *cancellatus*, in the region coding for testis-specific serine/threonine-protein kinase 1-like locus (99% identity: NC_022199). There was an insertion polymorphism (one bp) in the ddRAD locus compared to reference sequences in the NCBI gene bank.

Double digest RAD-seq using *Sbf*I and *Sph*I restriction enzymes generated over 1000 SNP markers that were used to produce a phylogeny of the ten species analysed. Principal component analysis of this dataset also clearly separated these ten species. From this dataset, 57 species-specific SNP markers were identified across the set of 10 tilapia species when 1–2 SNPs were allowed per ddRAD locus: larger numbers of species-specific markers were found when more SNPs were allowed per locus, or when the dataset was reduced to lower numbers of species. When screened on a larger set of individuals, many of these SNPs continued to show 100% discrimination, while in a minority of cases rare alleles were detected that were common to other species. While further development (*e*.*g*. larger sample sizes, testing on case studies) is required, this approach shows promise to investigate hybridisation and introgression in tilapias, both in the wild and in aquaculture, and could be applied to other species groups.

## Methods

### Ethics statement

All working procedures complied with the UK Animals Scientific Procedures Act (Parliament of the United Kingdom 1986). This research was carried out with the approval of the University of Stirling Animal Welfare and Ethical Review Body (AWERB).

### Biological materials

Fin samples were collected from 10 different tilapiine species. Efforts were made to source these only from populations with known histories and an absence of hybridisation. The *Oreochromis niloticus* samples consisted of two sub-species (*O*. *n*. *niloticus* and *O*. *n*. *cancellatus*) from three locations in each case; *O*. *aureus*, *O*. *mossambicus* and *Tilapia zillii* (Gervais: reclassification as *Coptodon zillii* proposed by Dunz & Schliewen, 2013^1^) comprised samples from two locations each, while *O*. *karongae* (Trewavas), *O*. *urolepis hornorum* (Norman), *O*. *andersonii*, *O*. *macrochir*, *Sarotherodon galilaeus* (Linnaeus) and *S*. *melanotheron* consisted of samples from one location each. Each originated from a single wild population (in some cases then maintained and bred in captivity) as far as could be ascertained. Samples were stored in 99% ethanol at −20 °C until needed. Details of samples and origins are listed in Table 1 and Supplementary Table S1. Sex ratios (see Supplementary Table S1) were approximately balanced as far as possible to minimise any potential bias due to sex-specific regions of the genome. Phenotypic sex data was available for 84% of the individuals used for the ddRADseq analysis; no phenotypic sex data was available for *O*. *andersonii*, *O*. *macrochir* or most of the sample of *T*. *zillii* from Ghana.

### Genomic DNA extraction

Total genomic DNA was extracted using the Realpure Genomic DNA Extraction Kit (Durviz S.L.) following the manufacturer’s protocol. An RNase incubation step was included to minimise RNA contamination, with each precipitated DNA sample being finally resuspended in 5 mM Tris, pH 8.5. Extracted DNA was quantified by spectrometry (NanoDrop ND 1000 Spectrophotometer, NanoDrop Technologies Inc.) and then by fluorimetry (Qubit® Fluorometer 2.0, dsDNA High Sensitivity assay kit; Invitrogen, ThermoFisher Scientific). Sample integrity was checked by agarose gel (0.8%) electrophoresis. Those samples comprising predominantly high molecular weight DNA and with both 260/280 and 260/230 OD ratios exceeding 1.8 were selected for use. Based on fluorimetry values samples were diluted to 7 ng/µL with 5 mM Tris, pH 8.5.

### Double Digest RAD library preparation and sequencing

Three libraries were constructed, including between 36–48 individuals in each (Supplementary Table S1). The ddRAD library preparation protocol was based on the methodology originally reported by Peterson *et al*.^25^, with modifications/refinements as described by Manousaki *et al*.^34^ and Brown *et al*.^35^. Briefly, for each library, individual DNA samples (21 ng–3 µL) were simultaneously digested with two high fidelity restriction enzymes: *Sbf*I (CCTGCA|GG recognition site), and *Sph*I (GCATG|C recognition site), both sourced from New England Biolabs (NEB), UK. Digestions were incubated for 40 min at 37 °C, using 0.25 U of each enzyme in 1× CutSmart Buffer (NEB), in a 6 µL reaction volume. The reactions were then cooled to c. 22 °C, 3 µL of a premade barcode/adapter mix was added to each digested DNA sample and incubated at 22 °C for 10 min. The adapter mix included individual-specific barcoded combinations of P1 (*Sbf*I-compatible) and P2 (*Sph*I-compatible) adapters at 6 nM and 72 nM concentrations respectively, in 1 × reaction buffer 2 (NEB). Adapters were compatible with Illumina sequencing chemistry. The barcoded adapters were designed such that adapter– genomic DNA ligations did not reconstitute RE sites, while residual RE activity limited concatemerisation of genomic fragments. The adapters included an inline five- or seven-base barcode for sample identification. Ligation was performed over 3.5 h at 22 °C by addition of a further 3 µL of a ligation mix including 4 mM rATP (Promega, UK), and 2000 cohesive-end units of T4 ligase (NEB) in 1× CutSmart buffer. The ligated samples were then heat denatured at 65 °C for 20 min and cooled to room temperature. Samples for a library were combined into a single pool. The pooled library sample was column-purified (MinElute PCR Purification Kit, Qiagen, UK), and eluted in 80 µL EB buffer (Qiagen, UK). Size-selection of fragments, ranging from 320 bp to 590 bp, was performed by agarose gel separation. Following gel purification (MinElute Gel Extraction Kit, Qiagen, UK), the eluted size-selected template DNA (60 µL in EB buffer) was PCR amplified (13–14 cycles PCR dependent on library; 24 separate 12.5 µL reactions, each with 1 µL template DNA) using a high fidelity Taq polymerase (Q5 Hot Start High-Fidelity DNA Polymerase, NEB). The PCR reactions were combined (300 µL total), and column-purified (MinElute PCR Purification Kit). The c. 50 µL eluate, in EB buffer, was then subjected to a further size-selection clean up using an equal volume of AMPure magnetic beads (Perkin-Elmer), to maximise removal of small fragments (less than c. 200 bp). Each final library was eluted in 19 µL EB buffer, QUBIT quantified and diluted to 10 nM stocks. Each library was sequenced in house on a separate Illumina MiSeq run (v2 chemistry, 300 cycle kit, 161 base paired-end reads; Illumina).

### Genotyping RAD-tags

The MiSeq generated reads were processed using a software pipeline designed specifically for RAD analysis, Stacks v1.46^36^. First, the *process_radtags* component was used to demultiplex the individual samples. During this step sequence reads with quality scores below 20, missing either restriction site or with ambiguous barcodes were also discarded. Barcodes were removed, and all sequences trimmed (3′ end) to be no greater than 148 bases long. For the purposes of this analysis paired-end reads were treated as separate loci, read 2 sequences being appended to read 1 sequence files. These sequences were assigned to RAD loci using both *de novo* and reference genome-based approaches (defaults parameters except where noted). The key parameter values employed to identify RAD loci for the *de novo* analysis (DBA) were: a minimum stack depth (m) of 6, a maximum of 2 mismatches allowed in a locus (M) in an individual and up to 4 mismatches between loci when building the catalog (n). For the reference-based analysis (RBA), the reads were aligned against *O*. *niloticus* genome using bowtie2, then a minimum stack depth (m) of 6 was used and included only those loci matched to the *O*. *niloticus* genome assembly Orenil1.1^37^, NCBI assembly GCF_000188245.2. Finally, the *populations* component of Stacks was used to export filtered data (polymorphic loci) for further analysis.

### Marker identification

Polymorphic loci were defined as RAD-tags with one or more SNPs. Shared loci were defined as polymorphic loci present in at least 75% of the samples, while species-specific loci were defined as polymorphic loci exhibiting no intraspecific polymorphism but showing interspecific polymorphism and present in at least 75% of each taxon. A marker was defined as one particular SNP at a locus.

### Phylogenetic analysis using SNP data

SNP data from filtered shared loci was combined into composite genotypes for each individual (n = 131). Phylogenetic trees were constructed with RAxML v8.0.0^38^. Maximum-likelihood phylogenetic trees were inferred using the GTR + CAT nucleotide substitution model^39^ and bootstrap support values estimated from 10,000 replicate searches of randomly generated trees. The best-scoring ML tree was visualised using FigTree v1.4.2.

### Physical mapping

The species-specific loci identified by RBA from the 10 species dataset were physically located in the *O*. *niloticus* genome and visualised using Genetic-Mapper v0.9^40^.

### Species discrimination analysis of SNP data

Principal Component Analysis (PCA) and Discriminant Analysis of Principal Components (DAPC) were carried out on the SNP data using R v3.3.2^41^ and an associated R/*adegenet* package v1.4-1^42^. PCA creates simplified models of the total variation within the dataset and DAPC identifies clusters of genetically related individuals^43^.

### PCR-based SNP genotyping

SNP assays were designed and manufactured for 24 species-specific SNP markers using KASP (Kompetitive Allele-Specific PCR) genotyping technology by LGC Genomics Ltd. (Supplementary Table S3). In most cases, these were derived from the set of species including *O*. *niloticus*, O. *mossambicus*, *O*. *aureus* and *O*. *u*. *hornorum*, but some markers were selected only to distinguish *O*. *mossambicus* from *O*. *niloticus* and *O*. *aureus* (see Results). For primer design to be feasible, the SNP of interest at a given locus needed to be at least 20 bp from the end of a given sequence. This allowed enough sequence for compatible primers to be designed. Each sample was genotyped in a 5 µL reaction volume using 8 ng DNA template dried in a 96 well white PCR plate (Starlab). The PCR cycling conditions (TAdvanced thermocycler, Biometra) were as follows: an initial denaturation at 94 °C for 15 min, 10 cycles at 94 °C for 20 s and touchdown 65 °C to 57 °C (dropping 0.8 °C each cycle) for 1 min followed by 35 cycles at 94 °C for 20 s and 57 °C annealing/amplification. Fluorescence signals were measured at 22 °C using a Techne Quantica® Real Time PCR Thermal Cycler (Techne) and genotypes assigned by allelic discrimination analysis using the Quansoft software v1.121.

### COI DNA barcoding

Approximately 655 bp of the COI gene from mitochondrial DNA was amplified from the 10 tilapia species (see Table 1), using primer pairs^44^ FishF2 -5′TCGACTAATCATAAAGATATCGGCAC3′ and FishR2 -5′ACTTCAGGGTGACCGAAGAATCAGAA3′. PCR was performed in 20 µL final volumes, each reaction containing 4 µL 5x Phusion HF buffer, 0.4 µL 10 mM dNTPs, 1 µL 10 µM FishF2 primer, 1 µL 10 µM FishR2 primer, 12.35 µL nuclease-free water, 0.25 µL Phusion DNA polymerase (2 units/µL; New England Biolabs) and 1 µL DNA template (c. 50 ng). The amplification conditions were: initial denaturation at 98 °C for 30 s followed by 33 cycles of 98 °C for 10 s, 59 °C for 30 s, 72 °C for 30 s and final extension at 72 °C for 10 min. The amplification products were purified by spin column following the manufacturer’s instructions (QIAquick PCR Purification kit). The purified samples were commercially sequenced bidirectionally (Sanger sequencing, GATC Biotech Ltd.). The COI sequences from ten tilapia species were aligned using Clustal Omega v1.2.2^45^, then a gene tree constructed based on a segment of 676 bases available for all individuals using RAxML and visualised using FigTree v1.4.2.

### Data access

All species names used are in accordance with The Catalogue of Life^46^. The raw sequence data from this study have been submitted to the EBI Sequence Read Archive (SRA) study PRJEB6979^47^.

## Supplementary information

Supplementary Information

Supplementary Table S1

Supplementary Table S2

Supplementary Table S3
