# Supplementary material for: Species-Specific Marker Discovery in Tilapia
Source: Sci Rep. 2019 Sep 10;9:13001. doi: 10.1038/s41598-019-48339-2 (PMC6737103; doi:10.1038/s41598-019-48339-2)
Supplement: Supplementary file 1 — Supplementary Information [file 41598_2019_48339_MOESM1_ESM.pdf]

Species-Specific Markers Discovery in Tilapia

Mochamad Syaifudin[1,2], Michaël Bekaert[1], John B. Taggart[1], Kerry L. Bartie[1], Stefanie Wehner[1,3], Christos Palaiokostas[1], M.G.Q. Khan[1,4], Sarah-Louise C. Selly[1], Gideon Hulata[5], Helena D'Cotta[6,7], Jean-Francois Baroiller[6,7], Brendan J. McAndrew[1], David J. Penman[1*]

Supplementary Information

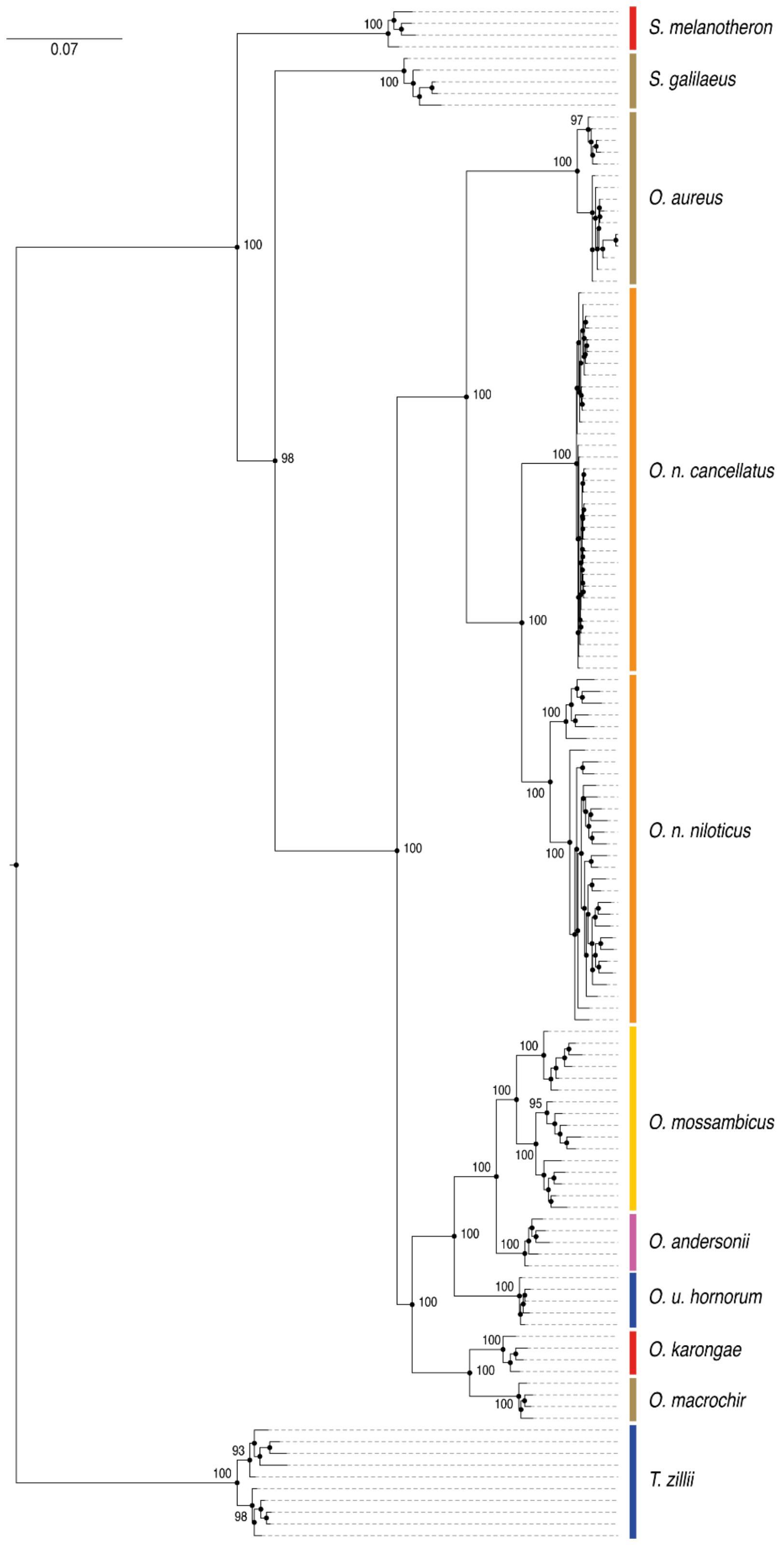

Figure S1. Phylogeny tree of tilapia species inferred from 1,371 common polymorphic SNP markers developed from *de novo*-based analysis (DBA) and rooted to *T. zillii*. All sequences were aligned, and the tree was constructed using RAxML (Randomized Axelerated Maximum Likelihood). The best ML bipartition tree is provided with bootstrap support when higher than 75%. STR, Stirling; AFI, Ein Feskha Israel; NSA, Natal South Africa.

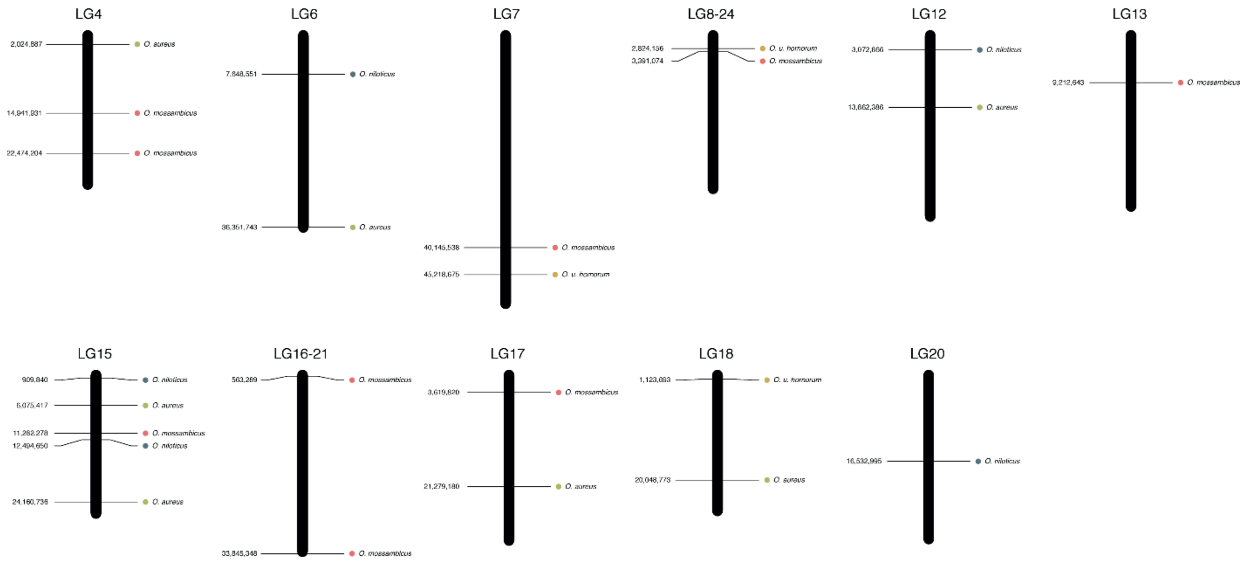

Figure S2. Distribution of 24 species-specific SNP markers (distinguishing among *O. aureus*, *O. mossambicus*, *O. niloticus* and *O. u. hornorum*) across the *O. niloticus* genome. LG: linkage group.
